# 肺癌患者手术前后凝血状态的变化

**DOI:** 10.3779/j.issn.1009-3419.2010.02.11

**Published:** 2010-02-20

**Authors:** 澄澄 徐, 向宁 付

**Affiliations:** 武汉 430030，华中科技大学同济医学院附属同济医院普胸外科 Department of General Thoracic Surgery, Tongji Hospital, Tongji Medical College, Huazhong University of Science and Technology, Wuhan 430030, China

**Keywords:** 肺肿瘤, 凝血功能, 高凝状态, 静脉血栓栓塞症, Lung neoplasms, Blood coagulation, Thrombophilia, Venous thromboembolis

## Abstract

**背景与目的:**

恶性肿瘤可引起机体高凝血状态, 可能导致血栓栓塞症的发生, 手术创伤则是重要的危险因素。本研究探讨肺癌患者手术前后凝血状态、变化及临床意义。

**方法:**

采用前瞻性对照研究方法, 选取普胸外科临床有效病例74例, 分为肺癌组和肺良性疾病组。观察患者术前和术后凝血酶原时间(prothrombin time, PT)、活化的部分凝血活酶时间(activated partial thromboplastin time, APTT)、血小板计数(platelet count, PLT)、D二聚体(D-dimer, D-D)及纤维蛋白原(fibrinogen, Fib)等指标反应的凝血功能状态、变化及临床表现, 比较组间组内之间的差别。

**结果:**

术前肺癌组较肺良性疾病组患者以及肺癌类型中腺癌患者较鳞癌患者术前Fib升高并高于正常值(*P* < 0.05)。肺癌组患者出现PT在术后第1天-第7天延长, APTT在术后第3天-第7天缩短, Fib在术后第3天-第7天、D-D在术后第1天-第7天升高, PLT在术后第1天、第3天较术前呈下降趋势而术后第5天、第7天有升高趋势, 与术前比较差异有统计学意义(*P* < 0.05)。肺癌组较肺良性疾病组患者术后第7天PT、D-D升高(*P* < 0.05)。术后肺癌组出现1例肺血栓栓塞症(pulmonary thromboembolism, PTE), 肺良性疾病组未出现静脉血栓栓塞症(venous thromboembolism, VTE)。

**结论:**

肺癌患者手术前后易出现高血凝状态并发VTE, 有必要行一定的干预措施避免VTE的发生。

## Abstract (en)

**Background and objective:**

Patients with malignant tumor are at high risk of thrombophilia, which contributes to thromboembolism.Surgical treatment is one of the critical risk factors.In this study, changes and clinical significances of blood coagulation of lung cancer patients pre- and post operation were investigated.

**Methods:**

A prospective, controlled study were carried out in 74 lung disease patients, who were divided into lung cancer group and benign lung disease group.In each group, pre-and postoperative changes in prothrombin time (PT), activated partial thromboplastin time (APTT), platelet count (PLT), D-dimer (D-D) and fibrinogen (Fib) and clinical performances were observed and compared in intra- and intergroups.

**Results:**

The concentration of Fib both in lung cancer group and its subgroup (adenocarcinoma of lung) increased, preoperative differences between benign lung disease group and subgroup (squamous cell carcinoma of lung) was significant (*P* < 0.05).PT(postoperative 1st to7th day) in lung cancer group prolonged, APTT (postoperative 3rd to7th day) reduced, Fib (postoperative 3rd to7th day) and D-D (postoperative 1st to 7th day) increased, PLT reduced on the 1st, 3rd day but then increased on the 5th, 7th day after operation, the difference between pre- and post-operation was significant (*P* < 0.05).D-D and PT in lung cancer group on the 7th day was longer than in benign lung disease group (*P* < 0.05).One pulmonary thromboembolism (PTE) case in lung cancer group occurred, while in benign lung disease group none venous thromboembolism (VTE) appeared.

**Conclusion:**

Patients with lung cancer are in high hypercoagulable state, and prone to VTE.It is necessary to take some interventions to avoid VTE.

普胸外科疾病中肺癌占肺部疾病的一大部分, 目前在全世界范围内所有恶性肿瘤中, 肺癌的死亡率也是最高。150多年前就有人提出恶性肿瘤和凝血状态有密切的关系^[1]^, 但对于肺癌患者手术前后凝血功能状态及变化的临床综合研究却很少。本研究采用前瞻性对照研究方法, 对35例肺癌和39例肺良性疾病患者手术前后凝血酶原时间(prothrombin time, PT)、活化的部分凝血活酶时间(activated partial thromboplastin time, APTT)、血小板计数(platelet count, PLT)、D二聚体(D-dimer, D-D)及纤维蛋白原(fibrinogen, Fib)进行对比研究, 从而了解凝血功能的变化及术后肺血栓栓塞症(pulmonary thromboembolism, PTE)及静脉血栓栓塞症(venous thromboembolism, VTE)的发生情况。

## 1 材料与方法

### 1.1 研究对象

入选对象为2009年3月-2009年7月在我院普胸外科接受肺部手术的患者。术后有明确病理诊断并根据病检分为肺癌组和肺良性疾病组。入选标准:患者无严重心肝肾胰腺疾患或造血功能障碍、出血倾向及出血性疾病; 无血管栓塞、动脉硬化、高血脂、冠心病等导致血栓形成的高危因素; 术前1个月内未服用过抗凝药物; 术后留院治疗1周以上; 肺癌患者能按国际TNM(1997年版)进行分期。术后1周内使用影响凝血功能药物患者不能入组。研究结束时肺癌组35例, 男性23例, 女性12例; 年龄46岁-72岁, 平均年龄56.5岁; 手术时间(262.42±72.7) min; 肺良性疾病组39例, 男性23例, 女性16例; 年龄35岁-76岁, 平均年龄54.5岁; 手术时间(254.2±74.1) min。在肺癌组中, 鳞癌18例, 腺癌14例, 其它类型3例; 有无区域淋巴结转移分别为16例和19例; TNM分期Ⅰ-Ⅱ期为15例, Ⅲ-Ⅳ期20例。两组患者性别、年龄和手术时间等无统计学差异。

### 1.2 研究方法和观察指标

采集术前及术后第1天、第3天、第5天、第7天清晨空腹静脉血。采用ACL TOP全自动血凝仪及配套试剂检测:PT正常值9 s-12 s; APTT正常值28 s-41 s; Fib正常值2.00 g/L-4.40 g/L; 采用ELISA法检测D-D正常值< 500 ng/mL; 采用雅培全自动血细胞分析仪及配套试剂检测PLT正常值100×10^9^/L-300×10^9^/L。

### 1.3 统计方法

采用SPSS 15.0统计学软件进行数据处理分析。所有统计检验都为双侧检验, *P* < 0.05为差异有统计学意义。计量数据用Mean±SD进行统计学描述, 采用均数*t*检验和方差分析(*ANOVA*)比较两组组内、组间的差别。计数数据采用频数(构成比)进行统计描述, 采用*χ*^2^检验比较两组差别。

## 2 结果

### 2.1 肺癌患者术前凝血功能的临床意义

肺癌类型中腺癌患者术前Fib均值高于正常值和鳞癌患者, 术前两组比较差异有统计学意义(*P* < 0.05)。而其它值组间比较差异无统计学意义(*P* > 0.05)。

术前凝血功能在肺癌是否区域淋巴结转移及分期间比较未见统计学差异(*P* > 0.05)(表 1)。

**1:** 术前凝血指标与肺癌类型、区域淋巴结转移、肺癌分期的关系 Relationship between preoperative haemotasis parameters and histology type, lymphonode metastasis, TMN stage of lung cancer

Items	PT (s)	APTT(s)	Fib(g/L)	D-D (ng/mL)	PLT (1×10^9^/L)
Histology type					
Squamous cell carcinoma	11.3±1.1	34.4±4.5	3.47±0.9	562.7±685.7	196.9±57.0
Adenocarcinoma	11.5±0.7	36.1±4.9	4.80±1.2^#^	276.8±142.0	261.9±107.4
Lymphonode metastasis					
N0	11.7±0.9	35.9±3.3	4.6±1.5	424.0±520.9	241.9±88.0
N1-3	11.1±0.9	34.4±6.2	4.2±1.3	317.3±183.7	230.6±95.4
TMN stage					
Ⅰ-Ⅱ	11.7±1.1	35.6±4.3	4.1±1.5	302.4±385.6	249.8±102.8
Ⅲ-Ⅳ	11.3±0.7	35.1±4.9	4.6±1.3	454.4±231.0	227.9±78.0
PT: prothrombin time; APTT: activated partial thromboplastin time; Fib: fbrinogen; D-D: D-dimer; PLT: platelet count; ^#^: *P* < 0.05 *vs* Squamous cell carcinoma.					

### 2.2 术前、术后凝血功能的变化

术前肺癌组较肺良性疾病组患者的Fib升高并高于正常值, 两者比较差异有统计学意义(*P* < 0.05)。而PT、APTT、D-D及PLT的平均值均在正常值范围之内, 术前两组比较差异无统计学意义(*P* > 0.05)。

肺癌组患者出现PT在术后第1天-第7天延长, APTT在术后第3天-第7天缩短, Fib在术后第3天-第7天升高, D-D在术后第1天-第7天升高, PLT在术后第1天、第3天较术前呈降低趋势, 而术后第5天、第7天有升高趋势, 与术前比较差异有统计学意义(*P* < 0.05)。

术后肺癌组较肺良性疾病组患者术后第7天PT、D-D升高, 组间比较差异有统计学意义(*P* < 0.05)(表 2)

**2:** 两组患者术前术后凝血指标的变化 The changes of perioperative haemotasis parameters in two groups

Groups	Pre-operation	The 1st day after operation	The 3rd day after operation	The 5th day after operation	The 7th day after operation
Lung cancer group					
PT	11.5±0.9	12.8±1.0^*^	12.7±1.3^*^	12.7±3.1^*^	13.1±3.4^*^
APTT	35.3±4.6	32.7±4.9^*^	32.6±4.8^*^	32.3±3.8^*^	33.3±4.1
Fib	4.4±1.4	4.1±1.2	6.5±4.9^*^	7.1±4.9^*^	6.8±5.2^*^
D-D	384.0±409.6	3428.7±2055.7^*^	1053.5±967.0^*^	875.6±550.7^*^	1773.2±878.5^*^
PLT	237.4±89.6	223.2±63.5	211.5±62.5	255.7±74.8	297.8±99.4^*^
Benign lung disease group					
PT	11.2±1.0	13.3±2.3	12.4±1.0	11.8±1.0	11.7±0.9^#^
APTT	34.9±7.0	33.2±7.3	30.9±5.2	32.1±4.7	31.9±3.2
Fib	3.8±1.2^#^	3.7±1.3	5.7±1.3	5.8±1.3	5.8±1.1
D-D	396.2±878.0	2624.1±1639.9	1118.6±1428.0	1060.1±773.8	1179.1±725.5^#^
PLT	260.9±97.8	246.5±78.5	219.1±71.0	248.8±61.7	300.4±77.2
^#^: *P* < 0.05 *vs* Lung cancer group; ^*^: *P* < 0.05 *vs* Preoperation.					

### 2.3 术后VTE的发生

肺癌组中1例患者术后第5天出现PTE(2.86%), 治疗后好转。肺良性疾病组未出现VTE。

## 3 讨论

### 3.1 肺癌患者术前凝血功能的异常的意义

大约有50%肿瘤患者及90%的肿瘤转移患者有凝血功能的异常, 继而出现VTE可能。国外报道肺癌患者VTE的发生率有4%-10%^[1]^。多方面因素可引起凝血功能的异常:肿瘤直接可通过释放组织因子或肿瘤促凝血素(cancer procoagulant)^[2, 3]^; 肿瘤间接作用通过产生细胞因子如肿瘤坏死因子-α(tumor necrosis factor-α, TNF-α)、白细胞介素-1(interleukin -1, IL-1)和血管内皮生长因子(vascular endothelial growth factor, VEGF)等或改变血管内皮细胞特性激活血小板; 肿瘤生长及转移的微环境等^[4]^。我们发现肺癌患者无论有无淋巴结转移或早晚期术前Fib都高于正常值, 且比良性疾病组升高(*P* < 0.05), 特别是与鳞癌患者相比, 腺癌患者的Fib升高(*P* < 0.05), 腺癌患者可能通过多途径促进Fib升高抑制Fib降解而影响凝血系统, 使机体处于高凝状态, 有发生VTE的危险。这也支持Chew等^[5]^和Blom等^[6]^报道的非小细胞肺癌中腺癌VTE发生率为鳞癌2倍-3倍结果。而其它凝血功能指标如PT、APTT、D-D和PLT等在肺癌类型、有无淋巴结转移或肿瘤早晚期方面未见明显的差别, 国内这方面已有研究^[7, 8]^, 但因样本量较小, 结论仍有所争议, 有待于多中心大样本的进一步研究证实。

### 3.2 肺癌患者术后凝血功能的改变

血液凝固由凝血因子参与、复杂的蛋白质酶解过程, 主要通过外源性和内源性两条途径。APTT、PT主要反映内、外源凝血系统各凝血因子的含量与活性。术后肺癌组患者术后PT延长, 凝血因子消耗, APTT缩短, 高凝状态(*P* < 0.05)。内源途径是血液和带负电荷的异物表面接触启动, 外源的始动凝血的组织因子(tissue factor, TF)来自组织, 其机制可能为胸外科手术本身及术后应激反应使广泛的血管内皮损伤暴露和产生大量TF及异物; 血管内皮细胞、单核细胞等又受到炎症和TNF-α、IL-1β等细胞因子的作用表达大量TF^[2, 9, 10]^, 激活相关凝血因子形成复合物再激活凝血酶原为凝血酶, 使纤维蛋白原分解成单体, 形成不溶于水的交联纤维蛋白多聚体, 转化为血栓前状态, 继而形成血栓。

纤溶系统的亢进也是高凝状态的重要的反应。Fib和D-D是反映体内存在纤溶亢进及高凝状态的较为特异性的指标。Fib由肝脏产生, 是血浆中含量最高的凝血蛋白, 被激活后转变为纤维蛋白多聚体, 具有极强的交联网络功能, 交联各种血细胞形成血凝块, 又可与血小板膜表面糖蛋白结合而介导血小板活化反应。D-D是交联的纤维蛋白在纤溶酶作用下裂解产生的一种特异性的代谢产物, 其存在能够表明体内有纤维蛋白形成和溶解, 即血栓形成和溶解。我们研究发现肺癌组患者术后Fib和D-D都有升高, 且持续至术后1周(*P* < 0.05), 表明术后机体存在纤溶亢进和高血凝状态, 持续时间长。主要机制可能为:胸外科手术及术后应激反应激活凝血系统, 形成大量血栓, 在这过程中机体必然产生大量Fib, 而血栓的纤溶亢进过程会产生D-D; 炎症和肿瘤等因素可激活内皮细胞和血小板产生纤溶抑制剂, 蛋白酶C抑制物、蛋白酶连接抑制素(PNI)和富组氨酸糖蛋白(HRG)等, 以抑制Fib的降解^[11]^。

PLT是骨髓成熟的巨核细胞胞浆裂解脱落的活性胞质小块, 具有粘附、聚集和释放的功能, 直接参与血栓形成并在血栓形成过程中起关键性作用。我们研究发现肺癌患者术后第1天-第3天PLT水平呈下降趋势, 可能与术中出血、术后渗出导致PLT的消耗以及前期输液量较多引起稀释性PLT减少有关, 但PLT消耗和产生活化是一个动态并此消彼长的过程, 术后第5天-第7天由于出血、渗出及输液量的减少, PLT水平呈升高过程(*P* < 0.05), 参与凝血功能紊乱的形成。PLT可通过术后炎症反应细胞间的直接作用或二磷酸腺苷、凝血酶、蛋白酶类和白介素等各种释放因子介导激活产生, 而激活的PLT有更强的粘附、聚集性和其他细胞如白细胞、血管内皮和肿瘤细胞作用性^[12]^, 使血液凝固性大大增强, 机体处于高凝状态, 导致微小血栓或血栓性疾病的发生率增加。

### 3.3 行抗凝治疗措施的必要性

通过上述讨论, 肺癌患者因肿瘤因素和紧张、焦虑等精神因素加之手术的创伤、疼痛等多种原因可使围手术期机体处于应激状态, 可引起凝血途径、纤溶系统和血小板功能的改变, 表现为凝血功能的紊乱, 纤维蛋白溶解亢进, 血小板粘附性及聚集性增高, 机体处于高血凝状态且持续时间术后1周以上。高凝状态是静脉血栓的形成主要因素之一^[13]^。胸外科术后患者VTE多发生于术后第3天-第7天, 发生率为7.4%-26%, PTE发生率为5%-6%^[14]^, 可导致预后不良。我们研究发现肺癌患者术前术后都出现高凝状态, 术后第7天肺癌组较肺良性疾病组患者部分凝血指标如PT、D-D仍升高(*P* < 0.05), 并在术后第5天肺癌组患者中出现1例PTE, 因此对肺癌患者根据临床检验指标围手术期行抗凝干预措施如药物或护理运动等有一定必要性。同时国外研究表明肿瘤患者抗肿瘤结合长期的抗凝治疗, 如LMWH, 可以有效延长患者的生存期^[15]^, 术后预防VTE的发生也优于短期的药物应用^[16, 17]^。但在国内肺癌术后长期使用抗凝药物还缺乏经验, 安全性和疗效还有待于进一步的评价。
